# Progression of Rare Inherited Retinal Dystrophies May Be Monitored by Adaptive Optics Imaging

**DOI:** 10.3390/life13091871

**Published:** 2023-09-05

**Authors:** Katarzyna Samelska, Jacek Paweł Szaflik, Barbara Śmigielska, Anna Zaleska-Żmijewska

**Affiliations:** 1 Department of Ophthalmology, Medical University of Warsaw, 02-091 Warsaw, Poland; 2 SPKSO Ophthalmic University Hospital, 00-576 Warsaw, Poland

**Keywords:** adaptive optics, cone dystrophy, cone–rod dystrophy, inherited retinal diseases, inherited retinal dystrophies, ocular imaging, photoreceptors, retina, retinal imaging, Stargardt disease

## Abstract

Inherited retinal dystrophies (IRDs) are bilateral genetic conditions of the retina, leading to irreversible vision loss. This study included 55 eyes afflicted with IRDs affecting the macula. The diseases examined encompassed Stargardt disease (STGD), cone dystrophy (CD), and cone–rod dystrophy (CRD) using adaptive optics (Rtx1™; Imagine Eyes, Orsay, France). Adaptive optics facilitate high-quality visualisation of retinal microstructures, including cones. Cone parameters, such as cone density (DM), cone spacing (SM), and regularity (REG), were analysed. The best corrected visual acuity (BCVA) was assessed as well. Examinations were performed twice over a 6-year observation period. A significant change was observed in DM (1282.73/mm${}^{2}$ vs. 10,073.42/mm${}^{2}$, p< 0.001) and SM (9.83 μm vs. 12.16 μm, p< 0.001) during the follow-up. BCVA deterioration was also significant (0.16 vs. 0.12, *p* = 0.001), albeit uncorrelated with the change in cone parameters. No significant difference in REG was detected between the initial examination and the follow-up (*p* = 0.089).

## 1. Introduction

### 1.1. Inherited Retinal Dystrophies

Inherited retinal dystrophies (IRDs) comprise a heterogeneous group of rare diseases that result in bilateral, irreversible vision loss. Although IRDs are genetic conditions, they represent a diverse group with varying inheritance patterns. The most-common IRD is rod–cone dystrophy (RCD), e.g., retinitis pigmentosa (RP), which primarily affects rods, with cone degeneration in RP originating in the outer retina.

Among the IRDs primarily affecting the macula, Stargardt disease (STGD) is the most-prevalent, with an incidence rate of approximately 1 in 10,000. Its manifestations include central vision loss, dyschromatopsia, and macular abnormalities, forming a “bull’s eye” pattern. This maculopathy results from abnormal accumulation of lipofuscin deposits in the central macula, with degeneration primarily affecting the retinal pigment epithelium (RPE) in the macular region. STGD symptoms typically emerge in the second decade of life. Cone–rod dystrophy (CRD) and cone dystrophy (CD) are IRDs less common than Stargardt disease, affecting approximately 1 in 30,000 to 1 in 40,000 individuals. CRD involves the degeneration of both cones and rods, while CD affects only cones; both of these conditions are progressive. These dystrophies primarily affect the macula and disturb central vision. Clinically, both CD and CRD present similarly, with bull’s eye maculopathy and bone spicule cells in the outer retina. The inheritance of the aforementioned IRDs may be autosomal dominant, autosomal recessive, X-linked, or unresolved [1,2,3].

Numerous genes have been identified with mutations that can lead to various IRDs, making genetic testing complex. The most-common gene mutation in STGD is autosomal recessive, involving the *ABCA4* gene, which encodes the ATP-binding cassette transporter protein found in photoreceptors. Over 900 *ABCA4* disease-causing sequences have been identified, most of which are autosomal recessive. Some of the *ABCA4* mutations are responsible for the occurrence of RCD, CD, and CRD [4,5]. Other genes implicated in STGD include *ELOVL4* and *PROM1*.

Pathogenic mutations leading to CD and CRD can be located in genes encoding proteins involved in photoreception and the phototransduction cascade, such as *OPN1MW* and *OPN1LW* (encoding cone opsins) or in variants of *PDE6C*, *PDE6H*, *CNGA3*, and *CNGB3* (encoding transporters involved in controlling cGMP intracellular concentration). Other mutations may occur in genes involved in photoreceptor outer segment morphogenesis, intraflagellar transport, and neurotransmitter release. Of the 32 gene mutations leading to CD or CRD, 6 exhibit a predilection for CD and 22 tend to result in CRD, but most mutations overlap. There are also forms of CD and CRD associated with mutations in *ABCA4*—the most-common gene affected in STGD—as well as mutations in *RPGR*, which most commonly leads to RCD [2]. A comprehensive list of genetic mutations leading to different IRDs can be found on RetNet (https://web.sph.uth.edu/RetNet/home.htm, accessed on 4 August 2023).

### 1.2. Diagnostic Methods in Inherited Retinal Dystrophies

A clinical diagnosis of an IRD is typically made based on the clinical image of the eye fundus, the patient’s history of vision loss in the first and second decades of life, and family history. Ancillary tests that aid in diagnosing IRDs include optical coherent tomography (OCT) of the macula, OCT angiography (OCTA), perimetry, electrophysiological testing (e.g., electroretinography (ERG)), fundus autofluorescence (FAF), and fluorescein angiography (FA) [5,6,7,8,9].

Monitoring of IRDs is challenging, and in most cases, universal guidelines for patient management are lacking.

The emergence of new methods for IRD evaluation is ongoing. These include emerging technologies such as adaptive optics (AO), adaptive optics OCT (AO-OCT), optoretinography, laser speckle flowgraphy, retinal oximetry, and functional magnetic resonance imaging [9,10]. Optoretinography maps the optical signal in response to a stimulus, whereas laser speckle flowgraphy enables real-time visualisation of circulation within ocular fundus structures. Retinal oximetry provides measurements of oxygen metabolism and oxygen diffusion from the choroidal circulation. Functional magnetic resonance imaging is a neuroimaging technique that records responses from the visual cortex. Adaptive optics, a ground-breaking technology, enables non-invasive imaging of retinal photoreceptors at the cellular level, making it a potent tool for visualising retinal pathologies. AO-OCT improves the quality of OCT imaging by correcting aberrations and mitigating quality degradation [9,10].

### 1.3. Therapy Perspectives in Inherited Retinal Dystrophies

Potential therapeutic techniques under development include gene supplementation, gene editing, antisense nucleotides, optogenetics, and stem-cell-based therapies. There is particular optimism for the successful treatment of IRDs with gene therapy [10]. The aim of these potential therapies is to slow down the degeneration of photoreceptors or improve their function.

The only currently available treatment for IRDs is gene therapy with voretigene neparvovec-rzyl (Luxturna^®^), approved by the Food and Drug Administration (FDA) in 2017 and the European Commission in 2018. Luxturna^®^ targets the *RPE65* gene, which is primarily responsible for Leber congenital amaurosis (LCA).

In STGD, a human treatment trial targeting the *ABCA4* gene was initiated, but it was later halted by the sponsor (ClinicalTrials.gov ID: NCT01367444). Another active trial involves optogenetic therapy incorporating the injection of multi-characteristic opsin in eyes with STGD (NCT05417126). The results of gene therapy trials in RCD targeting the *RPGR*, *RHO*, *PDE6A*, *PDE6B*, and *MCO* genes are still awaited (NCT03252847, NCT03116113, NCT03316560, NCT05748873, NCT04945772). To our knowledge, there are no human clinical trials targeting CD and CRD listed on ClinicalTrials.gov. However, several trials target the *CNGA3* and *CNGB3* gene mutations in another IRD, achromatopsia (ACHM) (NCT03278873, NCT02610582).

### 1.4. The Use of Adaptive Optics in Ophthalmology

Adaptive optics (AO) is an imaging technique initially developed for precise visualisation in astronomy, where it corrected atmospheric irregularities. The aberration-correcting system provides high-quality imaging of distant objects [11,12]. This high-quality visualisation is used in ophthalmology to evaluate the microstructures of the human retina with the precision to visualise a single cell. The AO retinal exam is quick and non-invasive. The assessment of the rods, cones, and retinal pigment endothelium cells has found its application in the management of diabetic retinopathy, age-related macular degeneration, glaucoma, and IRDs. Another possible application of AO in ophthalmology is the visualisation and measurement of the parameters of retinal microvessels: veins and arteries, which may be useful in diabetic retinopathy, prediabetes, hypertension, and glaucoma [13,14,15]. When combined with OCT, AO enables 3D imaging of retinal structures such as photoreceptors and retinal pigment epithelium [9].

The characteristics of cone mosaic parameters in a healthy eye have been defined [16]. Cone density in the healthy adult population averages 19,453/mm${}^{2}$; cone spacing is 7.96 μm; Voronoi analysis of cones (which is the percentage of hexagonal cells) is 46.7%.

**Figure 1:**
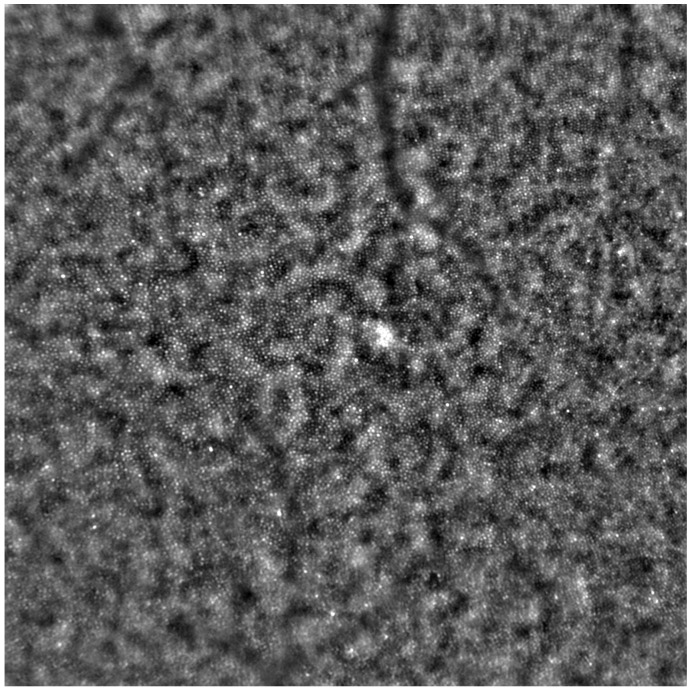
An adaptive optics image showing photoreceptors in a healthy eye (Rtx1™, Imagine Eyes, France). The photoreceptor mosaic appears intact (not disrupted) with individual photoreceptors visible as white and greyish spots.

**Figure 2:**
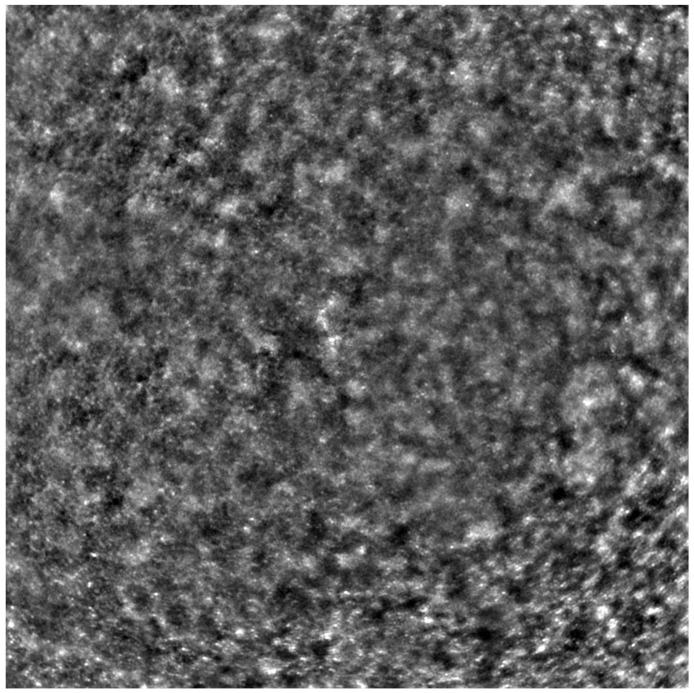
An adaptive optics image of the photoreceptors of an eye afflicted by cone dystrophy (Rtx1™, Imagine Eyes, France). Observe the cone disruption throughout the image with “dark spaces” apparent within the cone mosaic across different areas of the image.

**Figure 3:**
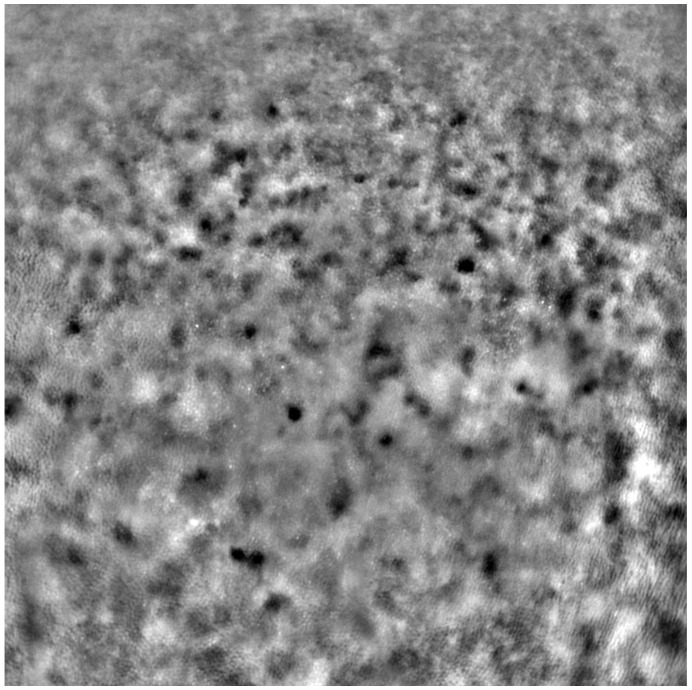
An adaptive optics image of photoreceptors in an eye affected by cone–rod dystrophy (Rtx1™, Imagine Eyes, France). Throughout the image, the cones are not clearly visible. Observe the “dark spaces” scattered within the cone mosaic across various regions of the picture.

**Figure 4:**
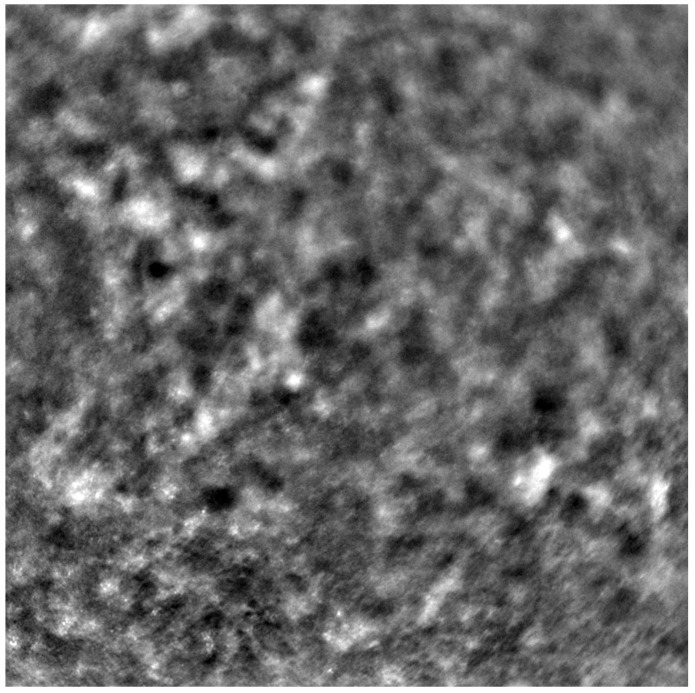
An adaptive optics image of the photoreceptors of the eye with Stargardt disease (Rtx1™; Imagine Eyes, France). The photoreceptor mosaic is disrupted, note the appearance “dark spaces” among the cone mosaic in various regions of the picture.

Adaptive optics retinal images depicting a healthy eye, CD, CRD, and STGD are presented in Figure 1, Figure 2, Figure 3 and Figure 4.

Figure 1 depicts the photoreceptor mosaic in a healthy eye. The image is taken paracentrally (2${}^{{}^{\circ}}$ superiorly) due to the limited ability of foveal image acquisition by Rtx™ [17]. By changing the focus point, the quality of an acquired image provides the assessment of photoreceptor parameters in parafoveal cones. The aberration and noise found in Figure 2, Figure 3 and Figure 4 are considered to be the result of poor fixation in eyes with impaired central vision in the course of macular disease. This issue has been addressed in our study. The assessment of factors predisposing for obtaining inadequate image quality was taken into consideration further in this article.

Cone mosaic disruption is an abnormality typical of IRDs. The cone and rod spacing is increased in IRDs compared to healthy retinas [18]. Additionally, poor image quality, likely resulting from inadequate fixation in eyes with low visual acuity, is a problem that, in some cases, makes image acquisition impossible [19,20]. In STGD, as well as in other IRDs, the “dark spaces” depicting areas of disrupted cone structure and abnormal cone reflectance have been described [21,22].

Rtx1™ (Imagine Eyes, Orsay, France) is an adaptive optics flood-illuminated ophthalmoscope (AOFIO) that uses infrared light (850 nm wavelength) with a 1.6 μm resolution. The image dimensions are 4${}^{{}^{\circ}}$× 4${}^{{}^{\circ}}$, which corresponds to approximately 1.2 mm × 1.2 mm of the retina. The observation of foveal cones in Rtx™ is limited, as mentioned above [17], so images of the extrafoveal retina are typically acquired. Researchers working with this device often bypass this limitation by choosing a parafoveal region for the analysis. The examined position can be selected (e.g., 2${}^{{}^{\circ}}$ superior, inferior, temporal, or nasal—as in our study). The image acquisition in a single position lasts 2–4 s, during which 40 individual images are acquired [13,23,24,25]. The Rtx1™ software provides two programs for data evaluation: AO Detect for the analysis of photoreceptors parameters and AO Detect Artery for the analysis of vessel parameters.

AO enables the visualisation of rods and cones, the two types of retinal photoreceptors. The parameters that adaptive optics can measure include: cone density, cone spacing, Voronoi analysis of hexagonal cells, reflectivity, regularity, metrics for the preferred orientation of cones, and local spatial anisotropy [6,16,17,26].

In our study, we analysed cone parameters: cone density (DM), cone spacing (SM), and cone regularity (REG). The abbreviations DM, SM, and REG are used by the AO Detect program and are further used in this article.

DM is expressed in 1/mm${}^{2}$; it is inversely correlated with SM, which measures the neighbour distance of each cone. REG (expressed in %) is important for providing the exclusion of inaccuracies caused by cell identification errors [17]. Our study focused on the use of AO in IRDs, specifically CD, CRD, and STGD, over a 6-year observation period. We performed AO retinal examinations twice over a 6-year observation period.

## 2. Materials and Methods

The study included 56 eyes from 28 patients who had been diagnosed with Stargardt disease (STGD) (38 eyes of 19 patients), cone dystrophy (CD) (10 eyes of 5 patients), or cone–rod dystrophy (CRD) (8 eyes of 4 patients). One eye, belonging to a female patient diagnosed with CD, was excluded from the analysis of photoreceptor parameters because it was not possible to obtain a good-quality image in any quadrant during the follow-up check. However, this eye was included in the analysis of factors that could potentially lead to incomplete data acquisition.

The examinations occurred in 2015 and were repeated in 2021, conducted at the Department of Ophthalmology, Medical University of Warsaw, in the SPKSO Ophthalmic University Hospital.

Each patient received his/her respective diagnosis of CD, CRD, or STGD through an evaluation that incorporated the clinical appearance of the eye fundus, FAF, AF, perimetry, and electrophysiological testing. Genetic testing was carried out in 20 patients, 13 of whom with STGD tested positive for *ABCA4* mutations. In the remaining patients, no mutation causing their conditions was found.

The exclusion criteria for the study encompassed other ocular pathologies such as glaucoma, cataract, previous ocular surgeries, history of uveitis, obesity (body mass index (BMI) > 30 kg/m${}^{2}$), and diabetes. Each participant, and parents of those under 18, provided written consent. This study adhered to the tenets of the Declaration of Helsinki and secured approval from the bioethics committee of the Medical University of Warsaw (KB/87/2015).

Before each examination, the best corrected visual acuity (BCVA) was checked using a Snellen chart, and the axial length of each eye was measured (LS 900; Haag-Streit; Koeniz, Switzerland). After mydriasis was induced using one drop of 1% tropicamide administered into each eye, the Rtx1™ (Imagine Eyes, France) test was performed. The acquired images were processed with the AO Detect program (version 3.4, also known as AO Image 3.4, Imagine Eyes, Orsay, France), providing numeric values for photoreceptor parameters: DM, cone density; SM, cone spacing; REG, cone regularity.

Each patient’s eyes were examined using Rtx1™ (Imagine Eyes, France), measuring four positions in each eye: 2${}^{{}^{\circ}}$ away from the fixation point in the superior, inferior, temporal, and nasal quadrants. The area selected for analysis was taken from within the examined frame, specifically in a location where the image quality was adequate for conducting a quantitative analysis.

Due to the poor quality of some scans, the image positions were not considered for subsequent statistical analysis. Instead, we computed the average values from all positions where image acquisition was possible.

Demographic data for patients at initial presentation are detailed in Table 1. The values of BCVA, DM, SM, and cone regularity are shown in Table 2 for the initial check and in Table 3 for the follow-up check.

Data underwent normal distribution testing using the Shapiro–Wilk test. For normal distributions, Student’s *t*-test was used to compare the mean values of independent variables. If the normality assumption was violated, we employed the non-parametric Mann–Whitney U-test to compare continuous variables between two groups of observations. For comparisons involving more than two groups (as in our case, where three diagnosis types existed), we used one-way ANOVA (for parametric tests) or the Kruskal–Wallis test (for non-parametric tests). These tests were followed by either the HSD Tukey’s post hoc test (ANOVA) or Dunn’s post hoc test (Kruskal–Wallis), with results adjusted using the Bonferroni method. For this analysis, we set the level of statistical significance to *p* = 0.05. All calculations were carried out in R (Version 4.0.2).

## 3. Outcomes

### 3.1. BCVA Change during the 6-Year Observation Period

There was a significant change in BCVA observed between the examinations in 2015 and 2021: 0.16 vs. 0.12 (*p* = 0.001), as shown in Table 4. This change was also observed when analysing the right eyes (*p* = 0.024, Table 5) and left eyes (*p* = 0.021, Table 6) separately.

BCVA was not found to differ significantly between diagnoses in the initial examination (*p* = 0.102, Table 2), nor in the follow-up (*p* = 0.111, Table 3). Moreover, the BCVA change over the 6-year follow-up was not correlated with the diagnosis (*p* = 0.705, Table 7).

### 3.2. Change in DM and SM during the 6-Year Observation Period

During the 6-year observation period, there was a significant decrease in DM (−3008.25/${\mathrm{mm}}^{2}$, SD = 3059.45/${\mathrm{mm}}^{2}$, *p* < 0.001) and an increase in SM (2.5 μm, SD = 4.13 μm, *p* < 0.001), as shown in Table 4. This significance was consistent when the calculations were performed separately for the right (*p* = 0.006 for DM and *p* = 0.002 for SM) and left eyes (*p* < 0.001 for both DM and SM), as shown in Table 5 and Table 6.

### 3.3. Correlation between Cone Parameters and Diagnosis

The lowest mean DM and highest mean SM were found in patients with STGD. Both the DM and REG parameters differed significantly between eyes with a different diagnoses (*p* = 0.032 for DM (Kruskal–Wallis test), *p* < 0.001 for REG (Kruskal–Wallis test) in the initial examination (Table 2). However, these findings were not confirmed in the follow-up exam (Table 3).

Regarding the change in cone parameters over the 6-year observation period, the highest DM change was noted in the CD group (−3697.39/${\mathrm{mm}}^{2}$), compared to the STGD (−3092.98/${\mathrm{mm}}^{2}$) and CRD (−1883.46/${\mathrm{mm}}^{2}$) groups. The highest SM change was observed in the STGD group (2.8 μm), compared to the CD (1.96 μm) and CRD (1.87 μm) groups. However, the intergroup difference in the DM and SM change was not significant (*p* = 0.338 for DM change, *p* = 0.308 for SM change), as shown in Table 7.

### 3.4. Change in REG during 6-Year Observation Period

No significant difference was observed between REG values from the initial and follow-up checks (86.44% vs. 84.37%, *p* = 0.089 for both eyes; 86.0% vs. 83.25%, *p* = 0.160 for right eyes only; 86.92% vs. 85.57%, *p* = 0.182 for left eyes only), as shown in Table 4, Table 5 and Table 6.

### 3.5. Correlation between BCVA Change, Cone Parameters Change, and Patient’s Sex

**Figure 5:**
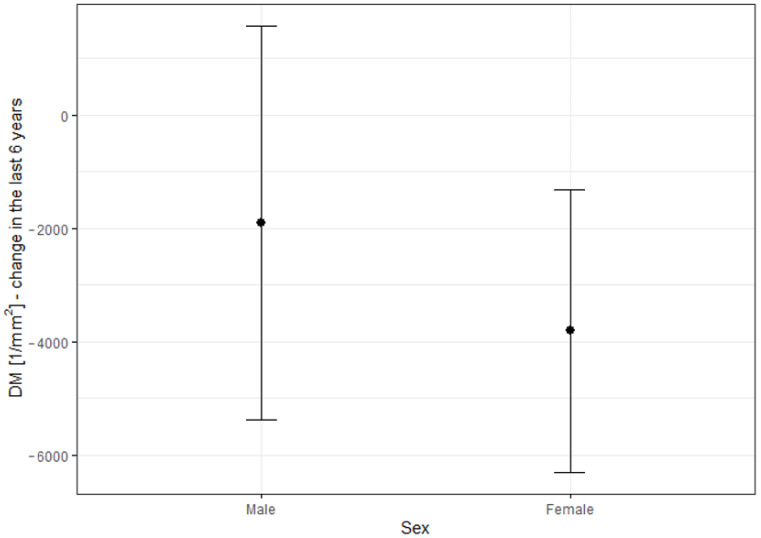
Difference in DM change over a 6-year observation period with respect to sex. DM: cone density (1/${\mathrm{mm}}^{2}$).

**Figure 6:**
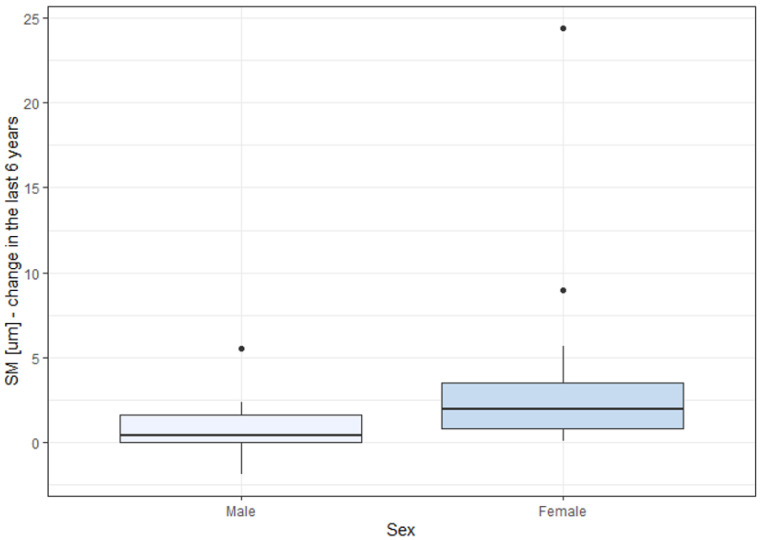
Difference in SM change over a 6-year observation with respect to sex. SM: cone spacing (μm).

The decrease in the DM and increase in the SM parameters were significantly higher in females than in males, as depicted in Figure 5 and Figure 6 and Table 8. Changes in DM and SM varied between the sexes. The mean DM change was (−1908.26/${\mathrm{mm}}^{2}$) for males and (−3804.8/${\mathrm{mm}}^{2}$) for females (*p* = 0.025). The mean SM change was 1.46 μm for males and 3.25 μm for females (*p* = 0.021). The changes in BCVA (−0.03 in males vs. −0.05 in females) and REG (−1.54% in males vs. −2.86% in females) were not significantly correlated with sex (*p* = 0.748 for BCVA change, *p* = 0.507 for REG change).

### 3.6. Correlation of DM Change over 6-Year Observation with BCVA and AO Parameters

The investigation aimed to ascertain whether the decrease in DM during the observation correlated with a functional parameter: BCVA. The Spearman analysis revealed no correlation between DM change from the initial measurement to the follow-up and either initial BCVA (*p* = 0.302) or BCVA change over the 6-year observation (*p* = 0.847), as illustrated in Table 9. However, a significant correlation was noted between DM change and REG deterioration over the observation period (*p* = 0.036). A robust correlation was also found between DM and SM change (correlation coefficient (r) = −0.856, *p* < 0.001), which stems from the definition of SM and DM.

### 3.7. Correlation of Cone Parameters with Patients’ Age

There was no observed correlation between patients’ age and either DM (*p* = 0.290) or SM (*p* = 0.185), as demonstrated in Table 10. Additionally, there was no correlation between DM change during the observation period and patients’ age (*p* = 0.223), as shown in Table 9.

### 3.8. Analysis of Factors Augmenting the Probability of Incomplete Data Acquisition

As previously mentioned, it was sometimes impossible to acquire an image of sufficient quality for analysis in all four examined quadrants. We conducted an analysis to identify the factors that increase the probability of incomplete data acquisition. The results of this analysis are presented in Table 11 and Table 12.

The ratio of incomplete data collection was 50% during the initial check and 33.9% during the follow-up. Factors such as patients’ age, sex, diagnosis, and BCVA were not found to be correlated with incomplete data acquisition. However, incomplete data collection was significantly associated with low DM (*p* < 0.001 during the initial check, *p* = 0.004 during the follow-up) and high SM (*p* < 0.001 during the initial check, *p* = 0.013 during the follow-up). Low REG was also identified as a factor contributing to incomplete data collection during the initial check (*p* < 0.001), but not during the follow-up (*p* = 0.357).

## 4. Discussion

Our study corroborates that the progression of CD, CRD, and STGD can be accurately tracked based on cone parameters with AO. We observed a deterioration in visual acuity (BCVA), a loss of cone density (DM), and an increase in cone spacing (SM) over a 6-year observation period. However, the changes in cone parameters did not correlate with the loss of BCVA in our study. We hypothesise that, due to the low standard deviation of the functional parameter used in our study (BCVA), the correlation of the morphological and functional parameters cannot be stated. The development of advancements in electrophysiology testing, such as multifocal electroretinography, as well as assessing the patients in early stages of disease, might be crucial for determining the real dependence of anatomical changes in photoreceptors on visual function.

Our goal was to discern whether differences existed in the adaptive optics cone parameters in eyes diagnosed with CD, CRD, and STGD. In both the initial exam of the study described in this article and in our other study [20], we found differences in DM, SM, and REG among the different diagnosis groups. However, these differences were not confirmed in the follow-up of our current study. We also did not find significant differences among the groups in terms of the DM, SM, and REG changes over time. This observation underscores the need for further longitudinal research on AO visualisation in eyes with IRDs, which would validate our findings.

The correlation between DM change and REG change may suggest a loss of quality in AO data with the progression of photoreceptor loss. However, since REG did not differ significantly between the initial check and the follow-up, we can infer that the quality of the obtained images of the cones was consistent across both examinations. More research is still required to confirm this correlation.

The highest BCVA was noted in the CRD group (0.32 on initial check, 0.28 on follow-up) and the lowest in the STGD group (0.13 on initial check, 0.09 on follow-up). The BCVA in the CD group was 0.17 on the initial check and 0.15 at the follow-up. Other clinical studies have confirmed that vision deterioration proceeds more slowly in CRD than in CD. Furthermore, nyctalopia is less common in patients with CRD [2,27]. However, in our study, BCVA deterioration over a 6-year period was not correlated with the diagnosis of CD, CRD, or STGD.

Changes in DM and SM over the six-year observation period were significantly higher in females than in males. This might be due to the higher percentage of females in the STGD group (57.1%, 16 women), compared to other groups. The percentages of females in the CD and CRD groups were 55.6% and 25%, corresponding to 5 and 2 women, respectively, indicating a small group size for reliable statistical analysis. To our knowledge, other studies have not reported a quicker progression of IRDs in females than in males. The observation of the greater prevalence of SM and DM changes in women with IRDs needs to be validated in future studies.

No inter-eye differences were noted in terms of cone density in our study, consistent with our previous study conducted on healthy eyes [16].

The rarity of inherited retinal dystrophies in the population constrained the number of patients in the study group. Several studies have compared AO imaging outcomes between eyes with IRDs and healthy eyes. For instance, Duncan et al. [28] contrasted 5 eyes with RP, 3 eyes with CRD, and 8 healthy eyes. Nakatake et al. [29] analysed 14 eyes with RP alongside 10 healthy controls. Additionally, Giannini et al. [30] included a range of conditions: 4 eyes with RP, 1 eye with best corrected macular dystrophy, 1 eye with occult macular dystrophy, 2 eyes with macular drusen, 4 eyes with nonproliferative diabetic retinopathy, and 20 healthy subjects. However, none of these studies provided longitudinal observations.

To our knowledge, no study other than ours has longitudinally assessed eyes with CD, CRD, or STGD. However, other reports on various IRDs do exist. For instance, a study on 16 eyes with RCD confirmed the correlation of AO changes with retinal thickness and findings in microperimetry, as well as a significant decline in cone spacing over a 3-year observation in the study group with no significant decline in healthy subjects [31].

Ueda-Consolvo et al. [32] carried out a study on 12 eyes of six patients with RP, aged 19–63, confirming the deterioration in cone density over a 2-year follow-up. BCVA was found to deteriorate in only one of six patients. This study did not include healthy controls. An observational case series by Ziccardi et al. [33] suggested the possibility of monitoring the progression of RP based on three probands over two years. A more-recent study [34] offered a short-term observation with AO imaging of eight patients with RCD and 10 healthy eyes, confirming the change of DM over a 6-month observation period in rod–cone dystrophy, but did not provide a longitudinal observation of healthy subjects.

Another publication reported the outcomes of imaging the eyes with RCDs [22], including patients who underwent neparovovec (Luxturna^®^) gene therapy. While the study did not report quantitative parameters such as DM and SM, it pointed out a crucial practical aspect of AO imaging in IRDs: the ability to assess the effects of treatments, like gene therapies. Evaluating photoreceptor parameters, along with visual assessment and other auxiliary tests, allows for a quantifiable analysis of the impacts of therapeutic interventions.

The progression of photoreceptor changes due to non-genetic pathology was documented by Potic et al. [35], where the study group consisted of patients post-retinal detachment repair, and the follow-up time was 3 months.

Differentiation between various types of IRD has been made possible through the use of AO across different genotypes. Mastey et al. [19] examined 9 subjects with ACHM, 7 of whom had a mutation in the *ATF6* gene, 1 in *CNGA3*, and 1 in *CNGB3*. In two of the patients, the acquisition of high-quality images was not feasible. Yet, they were able to discern a characteristic clear foveal cone mosaic in *CNGA3* and *CNGB3* patients, in contrast to patients with an *ATF6* gene mutation, where hyporeflective structures, possibly retinal pigment epithelium cells, were identified. The unique characteristics of the photoreceptor mosaic in retinitis-pigmentosa-GTPase-regulator (RPGR)-associated retinopathy and Stargardt disease and the differentiation between them have been reported [6].

A possible limitation to our study is the broad age range of the patients: 13–61 years, which could potentially impact the consistency of our study group. Nonetheless, in healthy eyes, the correlation between a patient’s age and cone density was not significant, as noted in our previous study [16]. There is no defined change rate in cone density and cone spacing. Foote et al. [31] found no change in cone spacing in healthy controls over a 3-year observation. Conversely, according to some authors, photoreceptor density appears to decrease over time. Our other study [15] describes the changes in AO parameters (mean cone density, cone spacing, cone regularity, and Voronoi analysis) over a two-year observation in healthy eyes and in patients with diabetes.

Another potential limitation of our study is the absence of a control group comprised of healthy eyes. The values of the photoreceptor parameters differed between our study and the study reporting a normative database of cone parameters of healthy eyes [16]. Longitudinal observations of eyes with IRDs and healthy controls are not commonplace, as outlined above. We believe that longitudinal studies comparing changes in DM and SM over time between eyes with IRDs and healthy eyes should be promoted.

One of the challenges encountered in adaptive optics (AO) imaging of eyes with inherited retinal diseases (IRDs) is the difficulty some eyes have in maintaining central fixation, which compromises the quality of the collected images. Additionally, when patients fixate eccentrically, the precise location of the captured image remains uncertain. Daich-Varela et al. [10] underscored the challenge of obtaining standardised AO images for each specific IRD.

In a previous study, we identified low cone density as a risk factor for incomplete data acquisition due to the poor quality of the obtained images, rendering quantitative analysis of photoreceptor parameters impossible [20]. These results align with those presented in the current article, as they are based on a similar cohort of patients. In our current study, the rate of full image acquisition failures ranged from 33.9% to 50%. We describe the correlation of low cone density and high cone spacing with the risk of data collection failure. The impact of low cone regularity as a predictor for incomplete data analysis remains uncertain, as it was not confirmed in a follow-up assessment. Both the current and previous analyses did not establish a correlation between visual acuity, sex, or age and the risk of poor image quality. We suspect that the researchers’ experience with the imaging device may impact the success of data collection, as the failure rate was higher during the initial assessment than during follow-up. Given the high percentage of eyes with IRDs, where obtaining high-quality images is often impossible, we propose considering average values of photoreceptor parameters collected from various quadrants rather than specific locations.

While the quality of imaging can be challenging in certain cases, it offers the ability to monitor disease progression, even in instances that are too advanced for successful standard monitoring with macular OCT, FAF, or electrophysiology testing.

## 5. Conclusions

We propose that adaptive optics is a dependable instrument in handling the complex task of tracking the progression of retinal diseases. Over the course of a 6-year observation, a significant change in the cone parameters DM and SM was recorded. The change in DM and SM was more pronounced in females than in males and appeared to be independent of diagnosis (STGD, CD, or CRD), BCVA, or age.

Our study group consisting of 55 eyes with IRDs, observed over a span of 6 years, is the largest and longest of its kind in terms of adaptive optics assessment of patients with IRDs. We anticipate that our findings will motivate other clinicians to incorporate this highly effective imaging tool into their practice. Presently, adaptive optics is predominantly used as a research method. We affirm that the data obtained with Rtx1™ are reliable, offering the potential for the long-term observation of disease progression. As the development of gene therapy in IRDs progresses, we believe that AO will serve as a valuable instrument for monitoring treatment efficacy.

## Figures and Tables

**Table 1:** Demographic data of the patients during the initial check (data collected in 2015). CD: cone dystrophy. CRD: cone–rod dystrophy. STGD: Stargardt disease.

	CD (*N* = 9)	CRD (*N* = 8)	STGD (*N* = 38)	Total (*N* = 55)
Age (years)				
Mean (SD)	38.11 (7.46)	44.75 (4.17)	35.89 (14.41)	37.18 (14.18)
Median	39	43.5	35	36.5
Range	30–55	41–67	13–61	13–67
Sex				
Female	5 (55.6%)	2 (25%)	24 (63%)	31 (56.4%)
Male	4 (44.4%)	6 (75%)	14 (37%)	24 (43.6%)
Eye				
Right	5 (55.6%)	4 (50%)	19 (50%)	28 (51%)
Left	4 (44.4%)	4 (50%)	19 (50%)	27 (49%)

**Table 2:** Characteristics of adaptive optics parameters with respect to diagnosis during the initial check (data collected in 2015). BCVA: best corrected visual acuity; DM: cone density (1/mm${}^{2}$), SM: cone spacing (μm); REG: cone regularity (%); CD: cone dystrophy; CRD: cone–rod dystrophy; STGD: Stargardt disease. The bold was used in all *p*-Values lower than 0.05 (=with statistical significance).

	All Patients	CD (*N* = 9)	CRD (*N* = 8)	STGD (*N* = 38)	*p*-Value (Test)
BCVA					
Mean (SD)	0.16 (0.19)	0.17 (0.13)	0.32 (0.32)	0.13 (0.16)	0.102
Median	0.08 (0.04–0.2)	0.2	0.1	0.06	(Kruskal–Wallis)
Range	(0.01–0.8)	0.02–0.4	0.04–0.8	0.01–0.7	
DM					
Mean (SD)	12,828.73	14,454.69	13,594.72	12,235.54	**0.032**
	(2618.96)	(2797)	(3067.63)	(2304.38)	(Kruskal–Wallis)
Median	13,018.33	14,523	14,434.62	12,513.33	
Range	7062–18,644.75	9368.67–18,644.75	8511.67–16,704	7062–18,080.5	
SM					
Mean (SD)	9.83 (1.01)	9.34 (1.04)	9.55 (1.12)	10.03 (0.95)	**0.034**
Median	9.62	9.18	9.09	9.75	(Kruskal–Wallis)
Range	8.1–12.72	8.1–11.61	8.62–11.61	8.22–12.72	
REG					
Mean (SD)	86.44 (4.44)	90.24 (2.52)	89.02 (2.24)	84.88 (4.38)	**<0.001**
Median	86.86	90.42	88.25	85.05	(Kruskal–Wallis)
Range	72.55–93.8	84.97–93.8	86.78–92.97	72.55–92.6	

**Table 3:** Characteristics of adaptive optics parameters with respect to diagnosis during the follow-up check (data collected in 2021). BCVA: best corrected visual acuity; DM: cone density (1/mm${}^{2}$); SM: cone spacing (μm); REG: cone regularity (%); CD: cone dystrophy; CRD: cone–rod dystrophy; STGD: Stargardt disease.

	All Patients	CD (*N* = 9)	CRD (*N* = 8)	STGD (*N* = 38)	*p*-Value (Test)
BCVA					
Mean (SD)	0.12 (0.16)	0.15 (0.09)	0.28 (0.34)	0.09 (0.09)	0.111
Median	0.05	0.16	0.06	0.05	(Kruskal–Wallis)
Range	0.01–0.8	0.01–0.25	0.04–0.8	0.01–0.4	
DM					
Mean (SD)	10,073.42	10,757.3	11,711.26	9523.2	0.208
	(3217.93)	(3839.17)	(3694.61)	(2861.4)	(Kruskal–Wallis)
Median	10,213.38	10,777.25	12,140	9123	
Range	3830–16,341.25	5627.33–16,341.25	4584.33–15,494.75	3830–15,499.88	
SM					
Mean (SD)	12.16 (4.19)	11.3 (2.46)	11.42 (4.11)	12.55 (4.58)	0.219
Median	11.08	10.5	9.91	11.34	(Kruskal–Wallis)
Range	8.59–35.08	8.59–15.51	8.86–21.18	9.11–35.08	
REG					
Mean (SD)	84.37 (6.96)	86.8 (4.35)	86.26 (4.48)	83.31 (7.77)	0.262
Median	86.03	88.47	86.61	85.66	(Kruskal–Wallis)
Range	60.66–96.77	77.46–91.31	79.05–92.46	60.66–96.77	

**Table 4:** Changes in adaptive optics parameters between the initial (2015) and follow-up (2021) checks. BCVA: best corrected visual acuity; DM: cone density (1/mm^2^); SM: cone spacing (μm); REG: cone regularity (%); CD: cone dystrophy; CRD: cone–rod dystrophy; STGD: Stargardt disease. The bold was used in all *p*-Values lower than 0.05 (=with statistical significance).

		Initial (*N* = 55)	Follow-Up (*N* = 55)	*p*-Value (Test)
BCVA	Mean (SD)	0.16 (0.19)	0.12 (0.16)	**0.001**
	Median	0.08	0.05	(Wilcoxon)
	Range	0.01–0.8	0.01–0.8	
DM	Mean (SD)	12,828.73 (2618.96)	10,073.42 (3217.93)	**<0.001**
	Median	13,018.33	10,213.38	(Wilcoxon)
	Range	7062–18,644.75	3830–16,341.25	
SM	Mean (SD)	9.83 (1.01)	12.16 (4.19)	**<0.001**
	Median	9.62	11.08	(Wilcoxon)
	Range	8.1–12.72	8.59–35.08	
REG	Mean (SD)	86.44 (4.44)	84.37 (6.96)	0.089
	Median	86.86	86.03	(Wilcoxon)
	Range	72.55–93.8	60.66–96.77	

**Table 5:** Changes in adaptive optics parameters between the initial and follow-up checks for right eyes only. BCVA: best corrected visual acuity; DM: cone density (1/${\mathrm{mm}}^{2}$); SM: cone spacing (μm); REG: cone regularity (%); CD: cone dystrophy; CRD: cone–rod dystrophy; STGD: Stargardt disease. The bold was used in all *p*-Values lower than 0.05 (=with statistical significance).

		Initial (*N* = 55)	Follow-Up (*N* = 55)	*p*-Value (Test)
BCVA	Mean (SD)	0.18 (0.23)	0.14 (0.17)	**0.025**
	Median	0.07	0.06	(Wilcoxon)
	Range	0.01–0.8	0.01–0.8	
DM	Mean (SD)	12,595.3 (2590.99)	10,357.02 (3246.84)	**0.006**
	Median	12,877	9396.5	(*t*-test)
	Range	7500–18,644.75	3830–15,499.88	
SM	Mean (SD)	9.97 (1.01)	12.4 (5.13)	**0.002**
	Median	9.82	11.31	(Wilcoxon)
	Range	8.1–12.18	8.85–35.08	
REG	Mean (SD)	86.00 (5.06)	83.25 (7.53)	0.160
	Median	86.38	85.66	(Wilcoxon)
	Range	72.55–93.8	60.66–96.77	

**Table 6:** Changes in adaptive optics parameters between the initial and follow-up checks for the left eyes only. BCVA: best corrected visual acuity; DM: cone density (1/${\mathrm{mm}}^{2}$); SM: cone spacing (μm); REG: cone regularity (%); CD: cone dystrophy; CRD: cone–rod dystrophy; STGD: Stargardt disease. The bold was used in all *p*-Values lower than 0.05 (=with statistical significance).

		Initial (*N* = 55)	Follow-Up (*N* = 55)	*p*-Value (Test)
BCVA	Mean (SD)	0.15 (0.15)	0.11 (0.16)	**0.021**
	Median	0.1	0.05	(Wilcoxon)
	Range	0.01–0.6	0.01–0.8	
DM	Mean (SD)	13,080.83 (2678.64)	9767.12 (3224.25)	**<0.001**
	Median	13,152.25	10,480.5	(T-test)
	Range	7062–18,080.5	4584.33–16,341.25	
SM	Mean (SD)	9.69 (1.02)	11.9 (2.95)	**<0.001**
	Median	9.44	10.53	(Wilcoxon)
	Range	8.21–12.72	8.59–21.18	
REG	Mean (SD)	86.92 (3.7)	85.57 (6.21)	0.182
	Median	85.95	86.17	(Wilcoxon)
	Range	77.22–92.6	66.67–95.84	

**Table 7:** A difference in change in BCVA, DM, SM, and REG over 6-year observation between CD, CRD, and STGD groups: both eyes. BCVA: best corrected visual acuity. DM: cone density (1/mm${}^{2}$). SM: cone spacing (μm). REG: cone regularity (%). CD: cone dystrophy. CRD: cone–rod dystrophy. STGD: Stargardt disease.

	All Patients (*N* = 55)	CD (*N* = 9)	CRD (*N* = 8)	STGD (*N* = 38)	*p*-Value (Test)
BCVA change					
Mean (SD)	−0.04 (0.1)	−0.03 (0.08)	−0.03 (0.14)	−0.04 (0.1)	0.705
Median	0	0	−0.04	0	(Kruskal–Wallis)
Range	(−0.4)–0.2	(−0.15)–0.1	(−0.3)–0.2	(−0.4)–0.04	
DM change					
Mean (SD)	−3008.25 (3059.45)	−3697.39 (1571.73)	−1883.46 (4462.61)	−3092.98 (2983.56)	0.338
Median	−3600.12	−3682.75	−1139.75	−3622	(Kruskal–Wallis)
Range	(−10,290.42)–3798.33	(−6004.92)–(−1358.5)	(−10,290.42)–3798.33	(−8683.33)–3185.83	
SM change					
Mean (SD)	2.5 (4.13)	1.96 (1.76)	1.87 (4.43)	2.8 (4.56)	0.308
Median	1.41	1.33	0.23	1.55	(Kruskal–Wallis)
Range	(−1.85)–24.39	0.38–5.66	(−1.85)–12.18	(−1.07)–24.39	
REG change					
Mean (SD)	−2.31 (7.66)	−3.44 (3.67)	−2.76 (4.36)	−1.89 (9.04)	0.475
Median	−1.82	−4.81	−1.82	0.05	(Kruskal–Wallis)
Range	(−27.74)–18.61	(−7.57)–2.26	(−11.55)–2.01	(−27.74)–18.61	

**Table 8:** Differences in BCVA, DM, SM, and REG changes over a 6-year observation period between males and females: both eyes. BCVA: best corrected visual acuity; DM: cone density (1/${\mathrm{mm}}^{2}$); SM: cone spacing (μm); REG: cone regularity (%); CD: cone dystrophy; CRD: cone–rod dystrophy; STGD: Stargardt disease. The bold was used in all *p*-Values lower than 0.05 (=with statistical significance).

	Males (*N* = 24)	Females (*N* = 31)	*p*-Value (Test)
BCVA change			
Mean (SD)	−0.03 (0.1)	−0.05 (0.1)	0.748
Median	0	0	(Mann–Whitney U)
Range	(−0.3)–0.2	(−0.4)–0.1	
DM change			
Mean (SD)	−1908.26 (3470.12)	−3804.8 (2492.9)	**0.025**
Median	−1528.75	−3741.33	(Mann–Whitney U)
Range	(−10,290.42)–3798.33	(−8683.33)–2622.88	
SM change			
Mean (SD)	1.46 (2.99)	3.25 (4.7)	**0.021**
Median	0.48	1.62	(Mann–Whitney U)
Range	(−1.85)–12.18	0.1–24.39	
REG change			
Mean (SD)	−1.54 (5.11)	−2.86 (9.12)	0.507
Median	−1.86	−1.79	(Mann–Whitney U)
Range	(−11.99)–6.91	(−27.74)–18.61	

**Table 9:** Correlation between DM change over a 6-year observation and initial parameters BCVA, DM, SM, REG, and age; BCVA change, SM change, and REG change. Initial BCVA: best corrected visual acuity at initial check (2015). Initial DM: cone density at initial check (2015) (1/${\mathrm{mm}}^{2}$). Initial SM: cone spacing at initial check (2015) (μm). Initial REG: cone regularity at initial check (2015) (%). BCVA change: difference in best corrected visual acuity between the initial check and follow-up. SM change: difference in cone spacing between the initial check and follow-up (μm). REG change: difference in cone regularity between the initial check and follow-up (%). The bold was used in all *p*-Values lower than 0.05 (=with statistical significance).

Variable	Correlation Coefficient (r)	*p*-Value	(Test)
initial BCVA	0.149	0.302	Spearman
initial DM	0.149	0.302	Pearson
initial SM	0.172	0.231	Spearman
initial REG	−0.157	0.276	Spearman
age (years)	−0.176	0.223	Pearson
BCVA change	0.028	0.847	Spearman
SM change	−0.856	**<0.001**	Spearman
REG change	0.297	**0.036**	Pearson

**Table 10:** Pearson’s correlation coefficient (r) between mean values of DM, SM, and age (data collected in 2021). DM: cone density (1/mm${}^{2}$), SM: cone spacing (μm).

Variable	Both Eyes (r)	*p*-Value	Right Eyes (r)	*p*-Value	Left Eyes (r)	*p*-Value
DM	−0.146	0.295	−0.328	0.110	−0.148	0.290
SM	0.197	0.157	0.316	0.124	0.185	0.185

**Table 11:** Descriptive characteristics concerning complete or incomplete data (with complete data indicating that 4 measurements provided an image suitable for analysis); data collected during the initial check. BCVA: best-corrected visual acuity; DM: cone density (1/mm${}^{2}$); SM: cone spacing (μm); REG: cone regularity (%); CD: cone dystrophy; CRD: cone–rod dystrophy; STGD: Stargardt disease. The bold was used in all *p*-Values lower than 0.05 (=with statistical significance).

		Incomplete Data (N=28)	Complete Data (N=28)	*p*-Value (Test)
Mean age (SD)		36.4(16.5)	37.9(11.4)	0.694
				(*t*-test)
Sex				
	Male	42.9%(N=12)	42.9%(N=12)	1
	Female	57.1%(N=16)	57.1%(N=16)	(chi-squared)
Diagnosis				
	CD	7.1%(N=2)	28.6%(N=8)	0.118
	CRD	14.3%(N=4)	14.3%(N=4)	(Fisher)
	STGD	78.6%(N=22)	57.1%(N=16)	
BCVA				
	Mean (SD)	0.15 (0.23)	0.21 (0.22)	0.112
	Median (IQR)	0.05 (0.03–0.12)	0.09 (0.05–0.29)	(Mann–Whitney U)
	Range	0.01–0.9	0.01–0.7	
DM				
	Mean (SD)	11,078.6 (2329.58)	14,003.66 (1905.81)	**<0.001**
	Median (IQR)	10,460.67 (9368.67–12,786.08)	13,805 (12,817.25–15,283.5)	(*t*-test)
	Range	7500–15,949	10.540–18,644.75	
SM				
	Mean (SD)	10.56 (0.98)	9.42 (0.65)	**<0.001**
	Median (IQR)	10.78 (9.84–11.21)	9.42 (8.99–9.82)	(*t*-test)
	Range	8.78–12.18	8.1–10.7	
REG				
	Mean (SD)	83.53 (5.56)	88.29 (3.08)	**<0.001**
	Median (IQR)	84.4 (79.47–87.03)	88.4 (86.2–90.6)	(*t*-test)
	Range	72.55–92.97	81.77–93.8	

**Table 12:** Descriptive characteristics concerning complete or incomplete data (with complete data indicating that 4 measurements provided an image suitable for analysis); data collected during the follow-up. BCVA: best-corrected visual acuity; DM: cone density (1/mm${}^{2}$); SM: cone spacing (μm); REG: cone regularity (%); CD: cone dystrophy; CRD: cone–rod dystrophy; STGD: Stargardt disease. The bold was used in all *p*-Values lower than 0.05 (=with statistical significance).

		Incomplete Data (N=19)	Complete Data (N=37)	*p*-Value (*Test*)
Mean age (SD)		39.63(14.3)	35.39(13.95)	0.360
				(*t*-test)
Sex				
	Male	47.4%(N=9)	40.5%(N=15)	0.839
	Female	52.6%(N=10)	59.5%(N=26)	(chi-squared)
Diagnosis				
	CD	21.1%(N=4)	16.2%(N=6)	0.838
	CRD	10.5%(N=2)	16.2%(N=6)	(Fisher)
	STGD	68.4%(N=13)	67.6%(N=25)	
BCVA				
	Mean (SD)	0.1 (0.12)	0.14 (0.18)	0.416
	Median (IQR)	0.05 (0.04–0.11)	0.06 (0.05–0.2)	(Mann–Whitney U)
	Range	0.01–0.4	0.01–0.8	
DM				
	Mean (SD)	8071.89 (2892.07)	10,884.85 (3011.82)	**0.004**
	Median (IQR)	7943.67 (5992–8886.5)	10.738 (9092.75–12,774.5)	(*t*-test)
	Range	4584.33–15,067	3830–16,341.25	
SM				
	Mean (SD)	13.17 (3.17)	11.75 (4.52)	**0.013**
	Median (IQR)	12.24 (11.36–13.91)	10.5 (9.8–11.76)	(Mann–Whitney U)
	Range	9.04–21.18	8.59–35.08	
REG				
	Mean (SD)	83.96 (7.48)	84.53 (6.83)	0.357
	Median (IQR)	84.76 (81.12–86.41)	86.17 (82.81–88.97)	(Mann–Whitney U)
	Range	66.67–96.77	60.66–92.46	

## Data Availability

Not applicable.

## Abbreviations

The following abbreviations are used in this manuscript:

ATP	Adenosine triphosphate
cGMP	Cyclic guanosine monophosphate
GTP	Guanosine triphosphate
IQR	Interquartile range
MDPI	Multidisciplinary Digital Publishing Institute
DOAJ	Directory of open access journals
LD	Linear dichroism
SD	Standard deviation
OR	Odds ratio
